# Trends in Prostate Cancer Incidence and Mortality Rates

**DOI:** 10.1001/jamanetworkopen.2024.56825

**Published:** 2025-01-27

**Authors:** Erin L. Van Blarigan, Meg A. McKinley, Samuel L. Washington, Matthew R. Cooperberg, Stacey A. Kenfield, Iona Cheng, Scarlett L. Gomez

**Affiliations:** 1 Department of Epidemiology and Biostatistics, University of California, San Francisco; 2 Department of Urology, University of California, San Francisco; 3 Greater Bay Area Cancer Registry, University of California, San Francisco

## Abstract

**Importance:**

Incidence of distant stage prostate cancer is increasing in the United States. Research is needed to understand trends by social and geographic factors.

**Objective:**

To examine trends in prostate cancer incidence and mortality rates in California by stage, age, race and ethnicity, and region.

**Design, Setting, and Participants:**

This cohort study used mortality data from the California Cancer Registry and California Department of Public Health's Center for Health Statistics, and incidence data from the National Cancer Institute Surveillance, Epidemiology, and End Results program and the US Census. The dataset for these analyses was released in April 2024. Participants included males residing in California between 2004 and 2021. Analyses were conducted from April to October 2024.

**Exposures:**

Stage at diagnosis, age, race and ethnicity, and region of California.

**Main Outcomes and Measures:**

The delay-adjusted incidence rates and mortality rates were calculated and age-adjusted to the 2000 US standard population. Annual percentage changes (APC) were calculated using NCI’s Joinpoint Regression Program.

**Results:**

Between 2004 and 2021, there were 387 636 prostate cancer cases (27 938 distant stage) and 58 754 prostate cancer deaths in California. In this study, 203 038 cases (52.4%) occurred among males aged 55 to 69 years, and 153 884 (39.7%) occurred among males 70 years or older. The distribution of race and ethnicity among cases was: 1031 American Indian or Alaska Native (0.3%); 31 366 Asian American, Native Hawaiian, and Pacific Islander (8.1%); 66 695 Hispanic or Latino (17.2%); 36 808 non-Hispanic Black (9.5%); 238 229 non-Hispanic White (61.5%); and 13 507 unknown or other races (3.5%). On average, the incidence of distant prostate cancer increased 6.7% (95% CI, 6.2% to 7.3%) per year between 2011 and 2021. By race and ethnicity, the APC ranged from 6.5% (95% CI, 4.2% to 13.4%) among Asian American, Native Hawaiian, and Pacific Islander males between 2011 and 2021 to 8.0% (95% CI, 6.9% to 9.5%) among Hispanic males between 2014 and 2021. In 9 of the 10 California regions, the incidence of distant prostate cancer increased by approximately 6% or more per year. Prostate cancer mortality rates declined 2.6% per year between 2004 and 2012 but plateaued between 2012 to 2021 (APC, 0.1%; 95% CI, −0.6% to 1.6%). The plateau in mortality occurred across ages, races and ethnicities, and regions.

**Conclusions and relevance:**

In this cohort study among California residents, the incidence of distant stage prostate cancer increased throughout the state between 2011 and 2021. Mortality rates plateaued between 2012 and 2021, ending previous decades of decline. Implementation of more effective prostate cancer screening strategies are critically needed.

## Key Points

**Question:**

How are prostate cancer incidence and mortality rates changing in California, and do trends vary by stage, age, race and ethnicity, or region?

**Findings:**

In this cohort study of males in California between 2004 and 2021, there were 387 636 cases of prostate cancer; the incidence rate of distant prostate cancer increased 6.7% per year, on average, between 2011 and 2021. On average, prostate cancer mortality rates declined 2.6% per year between 2004 and 2012 but plateaued between 2012 and 2021; trends in incidence and mortality were similar across age, race or ethnicity, and region.

**Meaning:**

These findings suggest that in the 2010s, distant stage prostate cancer increased and mortality rates plateaued throughout California.

## Teaser

This cohort study examines the trends in prostate cancer incidence and mortality rates in California by stage, age, race and ethnicity, and region of California.

## Introduction

Prostate cancer is in the most diagnosed and second-leading cause of cancer death among males in the US. While many prostate tumors are highly aggressive and eventually lead to death, many more are indolent and never metastasize. This heterogeneity in prostate cancer leads to challenges in screening and treatment. As the prostate specific antigen (PSA) screening test is not able to differentiate aggressive vs nonaggressive tumors, many males were diagnosed and treated for cancers that did not need intervention when PSA screening was first introduced and common in the US (approximately 1994 to 2008). On the other hand, in the absence of screening, prostate cancer is diagnosed with aggressive characteristics and usually too late for treatments to be curative.

The potential benefits and harms of PSA screening have resulted in fluctuating screening guidelines over the last 20 years. In 2008, the US Preventative Services Task Force (USPSTF) recommended against PSA screening for males older than age 75 years. In 2012, the recommendation for no PSA screening was extended to all males. In 2018, the guidelines were revised to recommend that males aged 55 to 69 years “discuss the possible benefits and harms of PSA screening with their health care [clinician] and make an individualized decision about whether to get screened.”^1^ It is unclear the extent to which shared decision-making is taking place and if population groups at higher risk of prostate cancer mortality (eg, non-Hispanic Black males) are being referred for screening. Thus, close monitoring of cancer surveillance data is needed to understand how the changing screening guidelines have impacted the incidence and mortality of prostate cancer across population groups.

The American Cancer Society reported a 4.5% annual increase in regional and distant-stage prostate cancer between 2011 and 2019.^2^ Among Black males, the population with the highest prostate cancer incidence and mortality rates, the incidence of distant-stage disease increased even more, at 5% per year, on average, between 2012 and 2018.^3^ Using data from the National Cancer Institute’s (NCI) Surveillance, Epidemiology, and End Results (SEER) program, Schafer et al^4^ reported similar trends across racial and ethnic groups, with the incidence of distant stage prostate cancer increasing 4% to 6% per year, on average, among Asian American or Pacific Islander, Hispanic, non-Hispanic Black, and non-Hispanic White males between 2011 and 2019. Nationally, prostate cancer mortality continued to decline through 2020, however the declining trend slowed substantially between 2013 and 2020 (from −6.2% to −1.3% among non-Hispanic Black males and from −3.4% to −0.7% among non-Hispanic White males). The decline in mortality plateaued among Asian American and Pacific Islander males and non-Hispanic White males in SEER registries after 2012.^4^

California is a racially, ethnically, and geographically diverse state with the expected greatest number of prostate cancer cases and deaths in 2023 of any state in the US.^2^ Given concerns regarding increasing incidence of distant stage prostate cancer, and potential differential changes in rates by demographic or regional factors, we examined trends in prostate cancer incidence and mortality through 2021 in California by stage, age, race and ethnicity, and region. We focused on regional variation within California, with the goal to determine if there are any regions not experiencing rapid rises in distant stage prostate cancer as well as identify regions with the highest need for intervention. This analysis includes 3 years of data after changes in the 2018 PSA screening guidelines.

## Methods

This cohort study used publicly available, deidentified data and was determined to not require institutional review board approval or informed consent at the University of California, San Francisco. We adhered to the Strengthening the Reporting of Observational Studies in Epidemiology (STROBE) reporting guideline.

### Data Sources and Variables

We obtained information on all invasive prostate cancer cases (*International Classification of Diseases for Oncology, Third Revision*, C61.9) diagnosed in California from January 1, 2004, to December 31, 2021 (the most recent available as of April 2024) from SEER. Information regarding prostate cancer cases, including year of diagnosis, age, tumor stage at diagnosis, county of residence, and race and ethnicity are routinely reported to the registries and data are abstracted from medical records. The April 2024 SEER Research Plus data release included data from 22 SEER registries (including the 3 California SEER registries) and provided delay factors specific to site, registry, race, ethnicity, stage, and year of diagnosis to adjust for a delay in reporting (or other inaccuracies).^5^ This database allows for more accurate reporting on recent incidence rates for prostate cancer because it takes into account expected corrections that are made over time as case documentation and quality control processes are completed.^6,7^ Data for race, ethnicity, age at diagnosis, county of residence at the time of diagnosis, and stage at diagnosis were abstracted for each case.

Deaths due to prostate cancer that occurred during the same period (2004 through 2021) were obtained from the California Cancer Registry and classified according to underlying cause of death.^8^ Information including race and ethnicity (typically recorded by a funeral director with input from an informant), age, and county of residence at death were obtained on each death event.^9^ California annual population counts, for both incidence and mortality, were obtained from SEER (Woods and Poole estimates).^10^

Race and ethnicity were classified as the following mutually exclusive groups: (2) American Indian or Alaska Native (available for individuals residing in purchased/referred care delivery areas only [PRCDA]); (2) Asian American, Native Hawaiian, and Pacific Islander; (3) Hispanic; (4) non-Hispanic Black; (5) non-Hispanic White; and (6) other or unknown race. A limitation of the race variable is that American Indian or Alaska Native individuals residing in non-PRCDA counties are classified as other or unknown race. As such, we report trends only among American Indian or Alaska Native cases residing in PRCDA counties. Forty of the 58 counties in California are PRCDA counties; 18 are non-PRCDA counties, including some of the larger counties (eg, Los Angeles, San Francisco, Alameda).

Using county of residence at the time of diagnosis or death, cases and deaths were classified into 1 of 10 regions developed by the California Census Office “based on their hard-to-count populations, like-mindedness of the counties, capacity of community-based organizations within the counties, and state census staff workload capabilities.”^11^ The regions included Superior California, North Coast, San Francisco Bay Area, Northern San Joaquin Valley, Central Coast, Southern San Joaquin Valley, Inland Empire, Los Angeles County, Orange County, and San Diego-Imperial. A list of counties by region is available in eTable 1 in Supplement 1.

### Statistical Analysis

Incident prostate cancer cases (delay-adjusted counts), death, and population counts were stratified by age, stage at diagnosis, race and ethnicity, and region of California (eTable 2 in Supplement 1). Prostate cancer annual delay-adjusted incidence rates and mortality rates, as well as 95% CIs (Tiwari modification), were calculated per 100 000 males and age-adjusted to the 2000 US standard population using the SEER Stat software, version 8.4.2 (National Cancer Institute).

Annual percentage change (APC) in rates and 95% CI were calculated for each age group, stage at diagnosis (for incidence), racial and ethnic group, and region using Joinpoint Regression Program version 5.0.2 (National Cancer Institute). Up to 3 joinpoints were allowed based on the number of data points (annual rates). Two-sided *P* values were used to test the hypothesis that the APC was 0. Following guidelines from the NCI, data from 2020 are omitted from incidence trend calculations to minimize the impact of pandemic-driven changes in cancer diagnoses on trend estimates; data from 2020 are included in estimation of mortality rate trends.^12^

The APC 95% CIs were calculated using the default empirical quantile method in Joinpoint.^13^ Trends were considered increasing or decreasing when the APC was statistically significant, determined by the inclusion or exclusion of 0 in the 95% CI. Annual rates were suppressed if the case counts or deaths were less than 16. APCs could not be calculated if any annual rate was suppressed; this occurred for distant stage prostate cancer incidence and mortality among the American Indian and Alaska Native population in PRCDA counties in California. As such, cases among American Indian and Alaska Native people were included in all analyses, but we could not report APCs in distant stage prostate cancer or mortality for this subgroup.

Lastly, to examine joint associations between race and ethnicity and region, we estimated trends in incidence of distant prostate cancer and prostate cancer mortality rates, by race and ethnicity, in the San Francisco Bay Area and Los Angeles County. These were the only 2 regions with enough cases to examine incidence and mortality trends for all 4 groups defined by race and ethnicity. We conducted these stratified analyses to explore the degree to which regional variation may reflect the differing racial and ethnic composition of the regions.

## Results

There were 387 636 cases of prostate cancer diagnosed in California between 2004 and 2021, of which 27 938 (7.2%) were distant stage at diagnosis; 58 754 deaths due to prostate cancer occurred in this period (eTable 2 in Supplement 1). In this study, 203 038 cases (52.4%) occurred among males aged 55 to 69 years and 153 884 (39.7%) occurred among males aged 70 years or older. Proportionally more distant stage cases and deaths occurred among males older than aged 70 years; 16 467 distant stage cases (58.9%) and 47 894 deaths (81.5%). The proportion of males from racial and ethnic minoritized groups was higher among distant stage cases compared with all prostate cancer cases. For total prostate cancer, 1031 tumors (0.3%) occurred among American Indian and Alaska Native males, 31 366 (8.1%) among Asian American, Native Hawaiian, and Pacific Islander males, 66 695 (17.2%) among Hispanic males, 36 808 (9.5%) among non-Hispanic Black males, 238 229 (61.5%) among non-Hispanic White males, and 13 507 (3.5%) among unknown or other race males. For distant stage prostate cancer, 90 tumors (0.3%) occurred among American Indian and Alaska Native males, 2507 (9.0%) among Asian American, Native Hawaiian, and Pacific Islander males, 5482 (19.6%) among Hispanic males, 3173 (11.4%) among non-Hispanic Black males, 16 571 (59.3%) among non-Hispanic White males, and 115 (0.4%) among unknown or other race males. For prostate cancer deaths, 493 (0.8%) occurred among American Indian and Alaska Native males, 3979 (6.8%) among Asian American, Native Hawaiian, and Pacific Islander males, 9325 (15.9%) among Hispanic males, 6401 (10.9%) among non-Hispanic Black males, 38 446 (65.4%) among non-Hispanic White males, and 110 (0.2%) among unknown or other race males.

**Figure 1.**
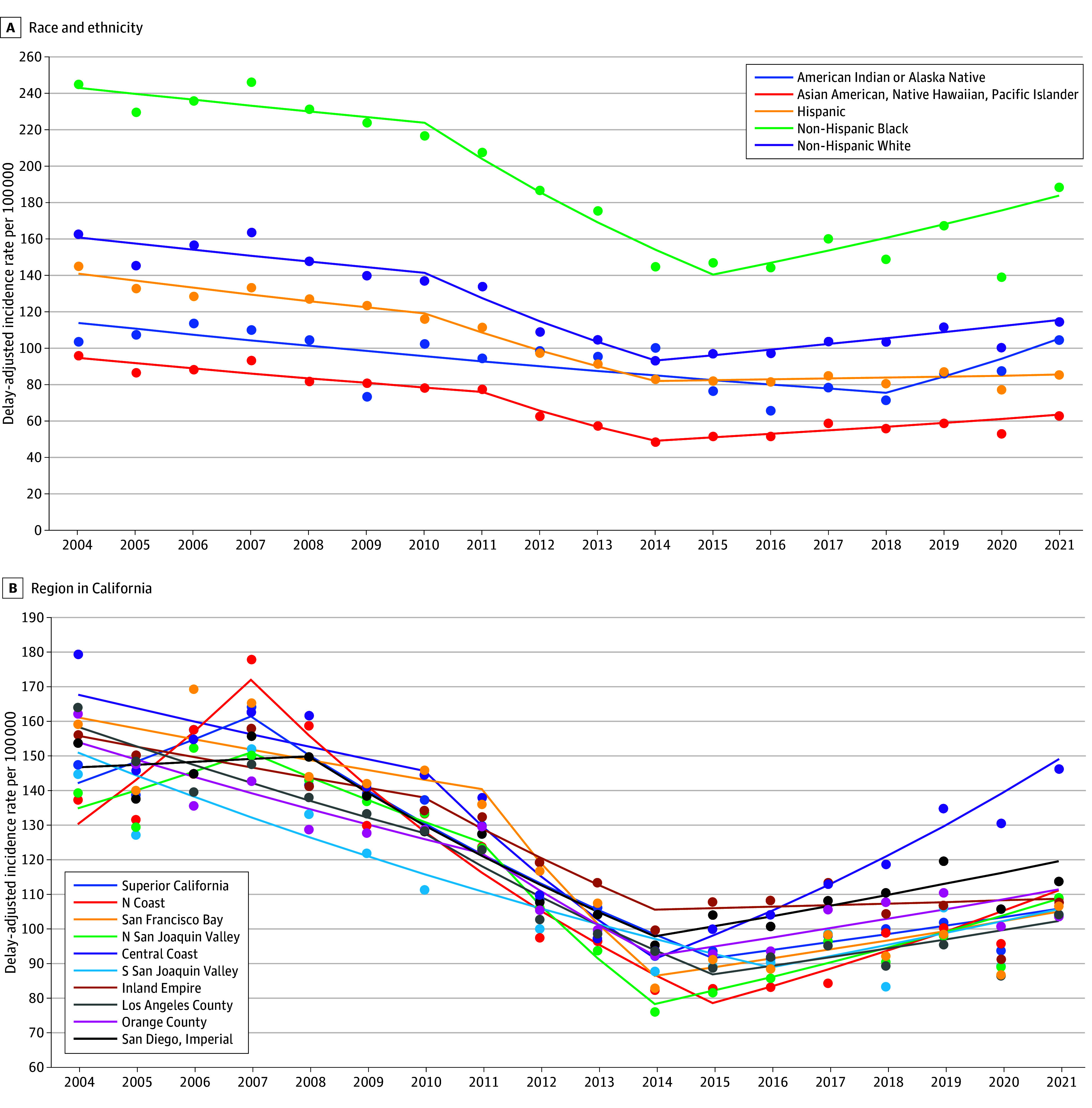
Delay-Adjusted Incidence Rates of Total Prostate Cancer (Per 100 000) in California (2004 to 2021) Dots indicate rate, and lines indicate trends. S indicates South; N, North.

Trends in the incidence of prostate cancer are shown in Figure 1A stratified by race and ethnicity and in Figure 1B stratified by region. Additionally, eTable 3 in Supplement 1 provides the APCs from the joinpoint models, overall and by stage, age, race and ethnicity, and region. The incidence of prostate cancer was stable in California between 2004 and 2010. On average, it decreased by 9.7% (95% CI, 7.0% to 13.2%) per year between 2010 and 2014 and increased by 2.6% (95% CI, 1.1% to 4.5%) between 2014 and 2021. This rise was driven by changes in regional and distant stage disease; there was no increase in the incidence of localized prostate cancer during the study period (eTable 3 in Supplement 1). Non-Hispanic Black males had the highest incidence rate, followed by non-Hispanic White males, Hispanic males, American Indian and Alaska Native males, and Asian American, Native Hawaiian, and Pacific Islander males. There was a statistically significant rise in overall prostate cancer incidence among non-Hispanic Black males (APC, 4.6%; 95% CI, 2.5% to 7.2%), Asian American, Native Hawaiian, and Pacific Islander males (APC, 3.7%; 95% CI, 2.4% to 5.3%), and non-Hispanic White males (APC, 3.1%; 95% CI, 1.4% to 5.3%) starting around 2014. There was also a marked increase in the incidence of prostate cancer among American Indian and Alaska Native males in PRCDA counties starting in 2018, but the APC was not statistically significant (APC, 11.5%; 95% CI, −0.8% to 18.2%). Regionally, the incidence of prostate cancer decreased across California between about 2007 to 2014, by 4% to 15%, on average per year, depending on region. After 2014, the incidence of prostate cancer increased by 3% to 7%, on average per year, in 5 of the 10 California regions and plateaued in the other 5. Notably, the incidence of prostate cancer was markedly higher in the Central Coast between approximately 2018 to 2021 compared with the other regions in California.

**Figure 2.**
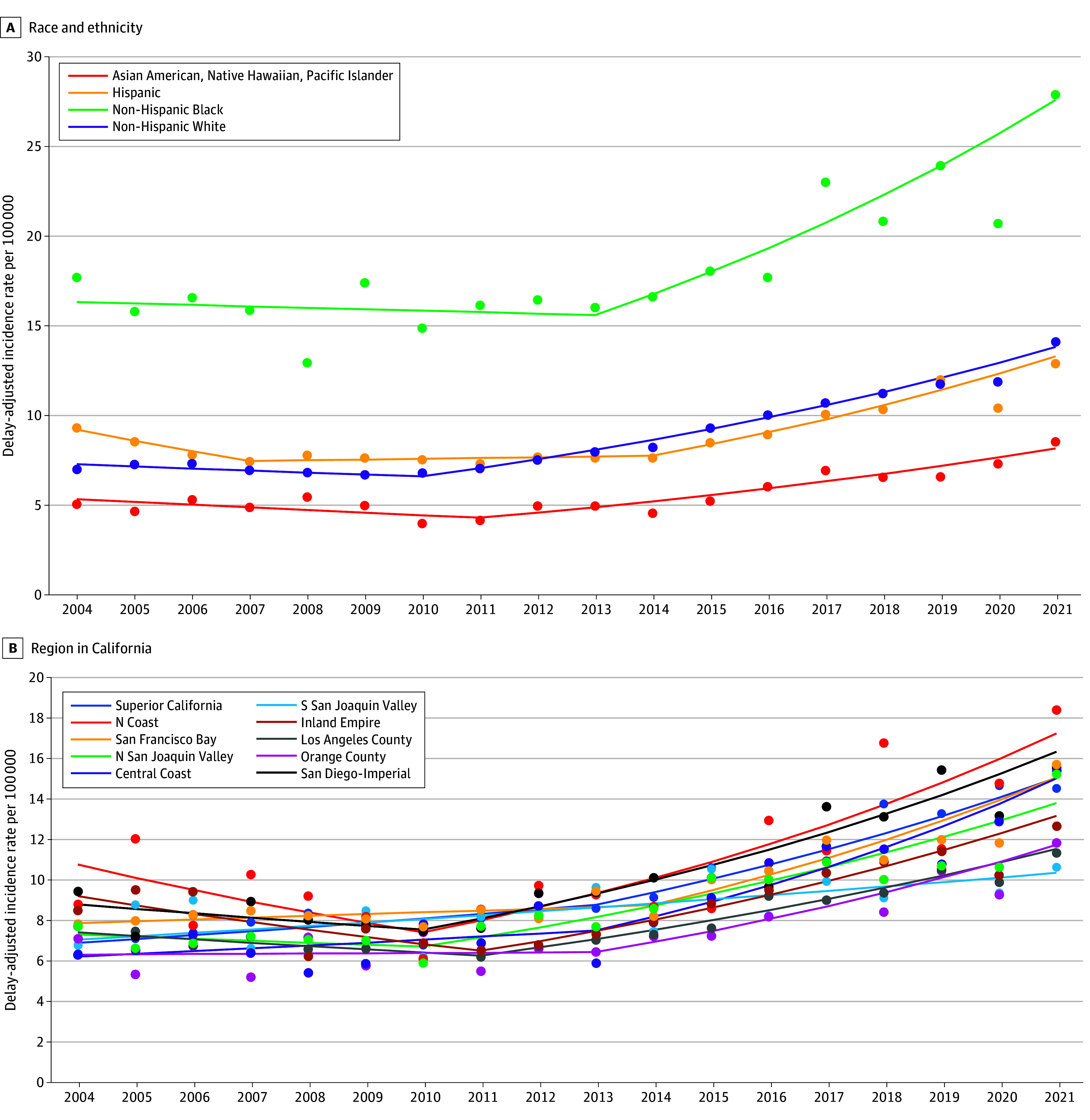
Delay-Adjusted Incidence Rates of Distant Stage Prostate Cancer (per 100 000) in California (2004 to 2021) Dots indicate rate, and lines indicate trends. S indicates South; N, North.

Trends in the incidence of distant stage prostate cancer are shown in Figure 2A stratified by race and ethnicity and in Figure 2B stratified by region. eTable 4 in Supplement 1 provides the APC from the joinpoint models by age, race and ethnicity, and region. The incidence of distant stage prostate cancer decreased between 2004 and 2011 and, on average, increased by 6.7% (95% CI, 6.2% to 7.3%) per year between 2011 and 2021. Non-Hispanic Black males experienced the highest incidence rates of distant stage prostate cancer; incidence rates were similar in recent periods for non-Hispanic White and Hispanic males and lowest among Asian American, Native Hawaiian, and Pacific Islander males. However, as with all prostate cancer cases, the patterns of change in incidence of distant stage disease were similar across age and racial and ethnic groups. All 4 racial and ethnic groups experienced a statistically significant increase in the incidence of distant stage prostate cancer in the most recent period, although the year that the rise began varied by group. Hispanic males experienced the largest rise (8.0% per year; 95% CI, 6.9% to 9.5%) between 2014 and 2021. This was followed by non-Hispanic Black males (7.4%; 95% CI, 5.1% to 11.4%) during a similar time frame (ie, 2013 to 2021), non-Hispanic White males (from 2010 to 2021: 6.9%; 95% CI, 6.4% to 7.5%), and Asian American, Native Hawaiian, and Pacific Islander males (from 2011 to 2021: 6.5%; 95%CI, 4.2% to 13.4%). The incidence of distant stage prostate cancer increased in all 10 California regions after 2010, with APC’s ranging from 2.3% (95% CI, 0.4% to 4.6%) per year in the Southern San Joaquin Valley from 2004 to 2021 to 9.1% (95% CI, 4.3% to 19.1%) per year in the Central Coast from 2013 to 2021.

**Figure 3.**
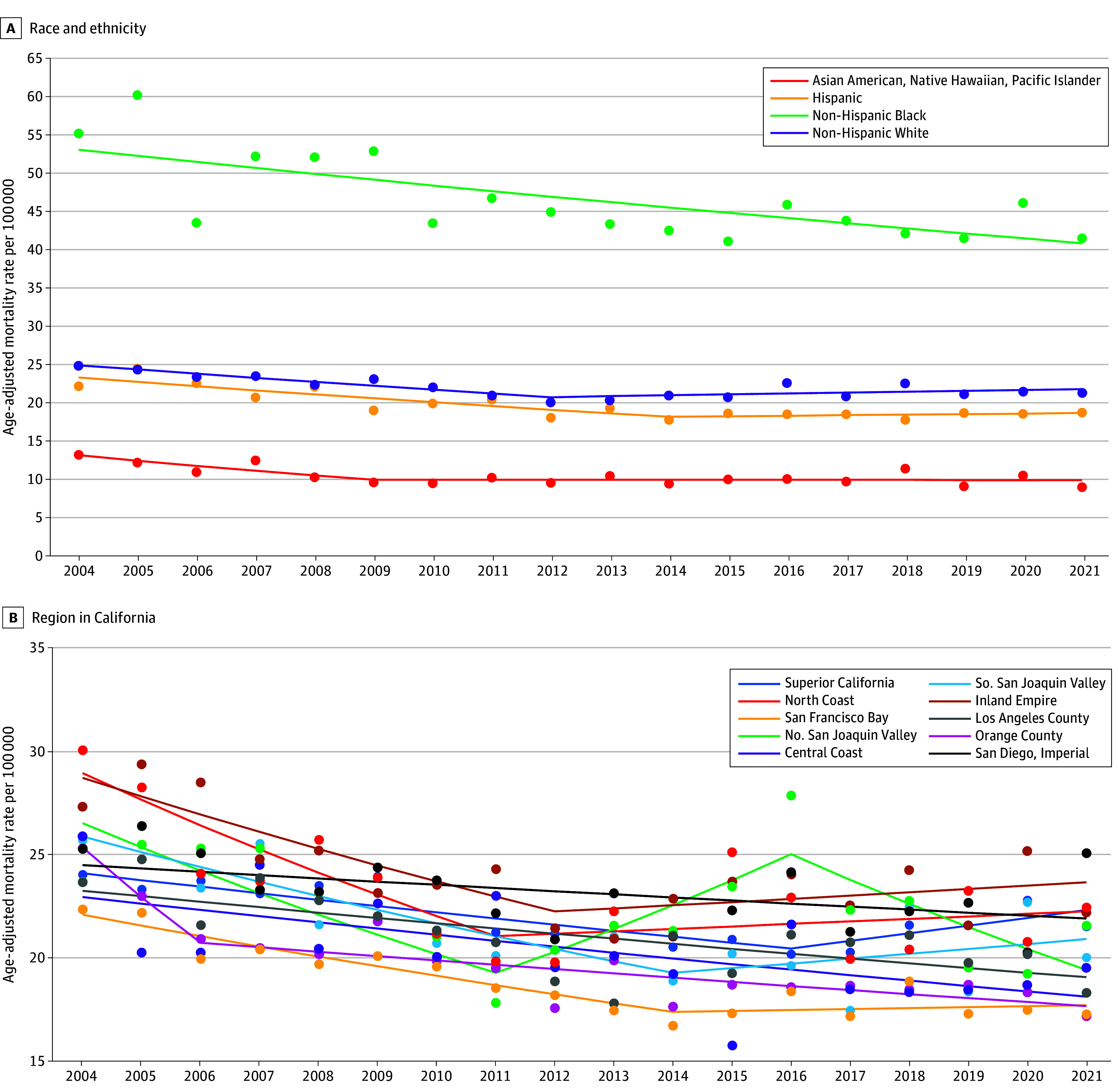
Prostate Cancer Mortality Rates Per 100 000 in California (2004 to 2021) Dots indicate rate, and lines indicate trends. S indicates South; N, North.

Trends in prostate cancer mortality are shown in Figure 3A by race and ethnicity and in Figure 3B by region. eTable 5 in Supplement 1 provides the APC in mortality by subgroups from the joinpoint models. Non-Hispanic Black males had the highest prostate cancer mortality rate between 2004 and 2021 in California, which is more than twice the mortality observed in any other racial or ethnic group. Prostate cancer mortality was lowest among Asian American, Native Hawaiian, and Pacific Islander males. The trends showed decreasing prostate cancer mortality among all groups initially, but mortality rates plateaued for Asian American, Native Hawaiian, and Pacific Islander males, Hispanic males, and non-Hispanic White males starting in 2009, 2012, and 2014, respectively. By age, the decline in mortality rates continued for males aged 55 to 69 years but plateaued for males aged 70 years or older between 2012 and 2021 (APC, 0.2; 95% CI, −0.5 to 1.3). Regionally, prostate cancer mortality rates were highest in the Inland Empire region and lowest in the San Francisco Bay Area. Over time, prostate cancer mortality decreased across California from about 2011 to 2014, after which the mortality rates plateaued in 7 of 10 California regions. The Northern San Joaquin Valley was the only region with a statistically significant increase in prostate cancer mortality between 2004 and 2021, which occurred between 2011 and 2016 (APC, 5.4%; 95% CI, 0.8% to 15.9%), but this was followed by a decrease in prostate cancer mortality between 2016 and 2021 (APC, −4.9%; 95% CI, −17.2% to −1.2%).

eFigures 1 to 4 in Supplement 1 show trends in the incidence rates of distant prostate cancer and prostate cancer mortality rates in the San Francisco Bay Area and Los Angeles County, stratified by race and ethnicity. These were the only 2 regions for which trends could be estimated in all 4 racial and ethnic groups. The incidence of distant prostate cancer increased in all combinations of race and ethnicity and region, although the rate of increase varied by region within racial and ethnic groups. For example, among Hispanic males, the incidence of distant prostate cancer had an average increase of 18.4% (95% CI, 5.4% to 26.4%) per year between 2018 and 2021 in the San Francisco Bay Area, and the incidence of distant stage disease among Hispanic males in Los Angeles County had an average increase of 7.1% (95% CI, 3.6% to 15.7%) per year between 2013 and 2021. Notably, the incidence of distant prostate cancer was higher in the San Francisco Bay Area than in Los Angeles County for all 4 racial and ethnic groups.

For mortality, there was no significant change over time among Asian American, Native Hawaiian, and Pacific Islander males in either the San Francisco Bay Area or Los Angeles County, and prostate cancer mortality had an average decrease of approximately 1.5% (95% CI, 0.6% to 1.8%) per year among non-Hispanic Black males in both regions (San Francisco Bay Area: −1.4%; 95% CI, −2.4% to −0.3%; Los Angeles County: −1.6%; 95% CI, −2.8% to −0.3%). However, regional differences occurred among Hispanic and non-Hispanic White males. Prostate cancer mortality decreased by 1.4% (95% CI, 0.8% to 2.0%) per year among Hispanic males in Los Angeles county between 2004 and 2021. There was no change over time in prostate cancer mortality among Hispanic males in the San Francisco Bay Area. On the other hand, prostate cancer mortality decreased 1.2% (95% CI, 0.6% to 1.8%) per year among non-Hispanic White males in the San Francisco Bay Area between 2004 to 2021. There was no change in prostate cancer mortality over this period among non-Hispanic White males in Los Angeles County.

## Discussion

The incidence of distant stage prostate cancer markedly increased throughout California in the 2010 decade, across ages, racial or ethnic groups, and regions. On average, the incidence of distant prostate cancer increased 6.7% per year between 2011 and 2021. Regionally, the APC in distant prostate cancer exceeded 6% in 9 of 10 California regions, reaching as high as a 9% increase, on average, per year between 2013 and 2021 in the Central Coast region. Overall, these data show a higher upward trend in the incidence of distant stage prostate cancer in California than previous nationwide reports.

The similar patterns in changing incidence across ages, races and ethnicities, and regions suggest that the change in PSA screening guidelines in 2012 that recommended against screening has thus far had a similar adverse impact on distant disease across population groups. Moreover, the updated 2018 guidelines do not appear to have yet impacted trends in prostate cancer incidence or mortality. Given that the 2018 guidelines recommend shared decision-making through patient-clinician conversations, there is concern these guidelines will exacerbate existing prostate cancer disparities for underserved populations with less access to health care as well as minoritized populations who may be less likely to engage in such discussions due to medical mistrust.^14,15^ It will be important to continue monitoring prostate cancer incidence trends by social factors as data emerge to understand how the 2018 guidelines have impacted different populations.

The prostate cancer mortality rate declined through 2012 in California but plateaued between 2012 and 2021. The only racial or ethnic group that continued to have a statistically significant decline in mortality through 2021 in California was non-Hispanic Black males, with an average 1.5% decrease in prostate cancer mortality per year. This was consistent with the report by Schafer et al^4^ that examined mortality data from 22 SEER registries through 2020. In that analysis, prostate cancer mortality had a significant declining trend through 2020 among non-Hispanic Black and Hispanic males but plateaued starting in 2013 and 2012 among non-Hispanic White and Asian American or Pacific Islander males, respectively. In California, among Asian American, Native Hawaiian, and Pacific Islander males, the joinpoint model showed a plateau in the mortality trend starting in 2009; among non-Hispanic White males, the plateau began in 2012, and among Hispanic or Latino males, the plateau in prostate cancer mortality occurred in 2014. It is remarkable that the prostate cancer mortality rate has continued to decline, albeit slowly, among non-Hispanic Black males despite a sharp increase in the incidence of distant stage disease in this population between 2015 and 2021. Racial disparities in prostate cancer mortality are driven by factors that occur before and after diagnosis. Indeed, data have shown that non-Hispanic Black men have comparable survival compared with non-Hispanic White men when prostate cancer management is standardized.^16,17^ It is possible that some of the excess mortality among non-Hispanic Black men (driven by factors other than higher incidence) improved sufficiently to counter rising mortality due to rising incidence during this period. This possibility is supported by data showing narrowing disparities in prostate cancer-specific mortality between non-Hispanic Black and non-Hispanic White men between 2005 to 2020.^18^ Nevertheless, given the rapid rise in distant stage disease (which has a 5-year survival of only 37%),^19^ it is likely that the prostate cancer mortality rate will plateau among non-Hispanic Black males in California in the coming years.

Across California, most regions tended to follow similar patterns in changes in prostate cancer incidence and mortality over time. For example, the incidence of distant stage disease was stable or decreased between 2004 and 2010 and increased starting in the early 2010s. This loosely corresponds to the changes in screening guidelines in 2008 and 2012. However, we also observed some regional differences, with implications for targeted cancer control efforts. The highest mortality rates from prostate cancer occurred in the Inland Empire region, which notably did not have the highest incidence of distant stage prostate cancer. This suggests efforts to improve access to guideline-based treatments among people with distant stage prostate cancer in the Inland Empire are needed. The next 2 regions with the highest mortality did have high incidence of distant stage disease: San Diego-Imperial and North Coast regions. The North Coast had one of the largest swings in incidence of total prostate cancer from 2007 to 2015 with an average 9.3% decrease per year, which may suggest quick adoption of the PSA screening guidelines to stop screening in that period. Unfortunately, this may have led to one of the largest subsequent rises in incidence of distant stage disease. The San Diego-Imperial region had among the highest incidence of both total and distant stage prostate cancer of any region in California. This region also had a high mortality rate but was 1 of the few regions to experience a statistically significant, albeit small, decline of less than 1% per year in the mortality rate from 2004 to 2021. The North Coast and San Diego-Imperial regions may be ideal settings to test implementation of contemporary evidence-based screening strategies.

Given large differences in prostate cancer incidence and mortality between racial and ethnic groups, and different demographic distributions between geographic areas, some of the regional variation may be driven by the racial and ethnic composition of the regions. To further explore this, we examined regional variation between the San Francisco Bay Area and Los Angeles County, within racial and ethnic groups. While our overall conclusions were consistent—that the incidence of distant stage prostate cancer was increasing, and mortality had plateaued—there were notable differences between regions within race and ethnicity. For example, distant stage prostate cancer increased 18.4% per year, on average, among Hispanic males (2018 to 2021) in the San Francisco Bay Area. In contrast, in Los Angeles County, the incidence of distant stage prostate cancer increased 7.1% per year, on average, for Hispanic males (2013 to 2021) (eFigure 2 in Supplement 1). Overall, trends in the incidence of distant stage prostate cancer were more similar across racial or ethnic groups in Los Angeles County, while they differed by race and ethnicity in the San Francisco Bay Area. Disparities in prostate cancer incidence and mortality are multifactorial, and include variations in screening, risk factors, prognostic factors, and disease management. Our data support the conclusion that policy discussions need to consider determinants of racial, ethnic, and geographic disparities to improve prostate cancer incidence and mortality.

### Strengths and Limitations

Strengths of this analysis include use of all prostate cancer cases and deaths in California between 2004 and 2021 and examination of rates by geographic region. Limitations include our inability to examine trends for distinct ethnic groups within the Asian American, Native Hawaiian, and Pacific Islander population in California, given lack of contemporary population estimates for Asian American, Native Hawaiian, and Pacific Islander ethnic groups. While the Asian American, Native Hawaiian, and Pacific Islander group has lower prostate cancer incidence and mortality compared to the other 3 groups reported here, the Asian American, Native Hawaiian, and Pacific Islander population is highly heterogeneous, and this aggregate grouping may mask important differences between specific ethnic groups. Second, race information for American Indian and Alaska Native individuals is only available in PRCDA counties; thus, we were not able to examine the incidence trends for this population statewide. There were also insufficient number of cases of distant stage disease or mortality to examine trends in these outcomes among American Indian and Alaska Native individuals in California. Lastly, the mortality data came from vital statistics and unfortunately stage at diagnosis was not available with these data. Consequently, we were unable to examine whether there has been a shift in the disease stage at detection among men who died from prostate cancer in this period.

## Conclusions

In this cohort study using data from California residents between 2004 and 2021, the incidence of distant stage prostate cancer was markedly rising across ages, racial and ethnic groups, and regions. Additionally, the mortality rate plateaued for most subgroups, despite previous years of decline. While the overall rising trends in incidence of distant stage prostate cancer were alarming across regions of California, we identified regions in need of improved access to guideline concordant care for distant disease (Inland Empire), as well as regions with particularly high need of improved screening strategies (eg, San Diego-Imperial, North Coast). Efforts to develop and implement evidence-based risk-stratified PSA screening are urgently needed to stop the rapid rise in distant stage prostate cancer and prevent the anticipated subsequent rise in prostate cancer mortality.

## Notes

Supplement 1. **eTable 1**. List of Counties by Region, California **eTable 2**. Number of Cases and Deaths Due to Prostate Cancer in California, by Race, Ethnicity, and Region (2004-2021) **eTable 3**. Trends in Prostate Cancer Delay-Adjusted Incidence Rates in California, Overall and by Stage, Age, Race and Ethnicity, and Region (2004-2021) **eTable 4**. Trends in Delay-Adjusted Incidence Rates for Distant Stage Prostate Cancer in California, Overall and by Age, Race and Ethnicity, and Region (2004-2021) **eTable 5**. Trends in Prostate Cancer Mortality Rates in California, Overall and by Age, Race, and Ethnicity, and Region (2004-2021) **eFigure 1.** Delay-Adjusted Incidence Rates of Total Prostate Cancer and Age-Adjusted Prostate Cancer Mortality Rates (Per 100,000) Among Asian American, Native Hawaiian, and Pacific Islander Males in Los Angeles County and the San Francisco Bay Area (2004-2021) **eFigure 2.** Delay-Adjusted Incidence Rates of Total Prostate Cancer and Age-Adjusted Prostate Cancer Mortality Rates (Per 100,000) Among Hispanic or Latino Males in Los Angeles County and the San Francisco Bay Area (2004-2021) **eFigure 3.** Delay-Adjusted Incidence Rates of Total Prostate Cancer and Age-Adjusted Prostate Cancer Mortality Rates (per 100,000) Among Non-Hispanic Black Males in Los Angeles County and the San Francisco Bay Area (2004-2021) **eFigure 4.** Delay-Adjusted Incidence Rates of Total Prostate Cancer and Age-Adjusted Prostate Cancer Mortality Rates (per 100,000) Among Non-Hispanic White Males in Los Angeles County and the San Francisco Bay Area (2004-2021)

Supplement 2.
Data Sharing Statement
