# Supplementary material for: Trends in Prostate Cancer Incidence and Mortality Rates
Source: JAMA Netw Open. 2025 Jan 27;8(1):e2456825. doi: 10.1001/jamanetworkopen.2024.56825 (PMC11774093; doi:10.1001/jamanetworkopen.2024.56825)
Supplement: Supplement 1. — eTable 1. List of Counties by Region, California eTable 2. Number of Cases and Deaths Due to Prostate Cancer in California, by Race, Ethnicity, and Region (2004-2021) eTable 3. Trends in Prostate Cancer Delay-Adjusted Incidence Rates in California, Overall and by Stage, Age, Race and Ethnicity, and Region (2004-2021) eTable 4. Trends in Delay-Adjusted Incidence Rates for Distant Stage Prostate Cancer in California, Overall and by Age, Race and Ethnicity, and Region (2004-2021) eTable 5. Trends in Prostate Cancer Mortality Rates in California, Overall and by Age, Race, and Ethnicity, and Region (2004-2021) eFigure 1. Delay-Adjusted Incidence Rates of Total Prostate Cancer and Age-Adjusted Prostate Cancer Mortality Rates (Per 100,000) Among Asian American, Native Hawaiian, and Pacific Islander Males in Los Angeles County and the San Francisco Bay Area (2004-2021) eFigure 2. Delay-Adjusted Incidence Rates of Total Prostate Cancer and Age-Adjusted Prostate Cancer Mortality Rates (Per 100,000) Among Hispanic or Latino Males in Los Angeles County and the San Francisco Bay Area (2004-2021) eFigure 3. Delay-Adjusted Incidence Rates of Total Prostate Cancer and Age-Adjusted Prostate Cancer Mortality Rates (per 100,000) Among Non-Hispanic Black Males in Los Angeles County and the San Francisco Bay Area (2004-2021) eFigure 4. Delay-Adjusted Incidence Rates of Total Prostate Cancer and Age-Adjusted Prostate Cancer Mortality Rates (per 100,000) Among Non-Hispanic White Males in Los Angeles County and the San Francisco Bay Area (2004-2021) [file jamanetwopen-e2456825-s001.pdf]

Supplemental Online Content

Van Blarigan EL, McKinley MA, Washington III SL, et al. Trends in prostate cancer incidence and mortality rates. *JAMA Netw Open*. 2024;8(1):e2456825. doi:10.1001/jamanetworkopen.2024.56825

eTable 1. List of Counties by Region, California
eTable 2. Number of Cases and Deaths Due to Prostate Cancer in California, by Race, Ethnicity, and Region (2004-2021)
eTable 3. Trends in Prostate Cancer Delay-Adjusted Incidence Rates in California, Overall and by Stage, Age, Race and Ethnicity, and Region (2004-2021)
eTable 4. Trends in Delay-Adjusted Incidence Rates for Distant Stage Prostate Cancer in California, Overall and by Age, Race and Ethnicity, and Region (2004-2021)
eTable 5. Trends in Prostate Cancer Mortality Rates in California, Overall and by Age, Race, and Ethnicity, and Region (2004-2021)
eFigure 1. Delay-Adjusted Incidence Rates of Total Prostate Cancer and Age-Adjusted Prostate Cancer Mortality Rates (Per 100,000) Among Asian American, Native Hawaiian, and Pacific Islander Males in Los Angeles County and the San Francisco Bay Area (2004-2021)
eFigure 2. Delay-Adjusted Incidence Rates of Total Prostate Cancer and Age-Adjusted Prostate Cancer Mortality Rates (Per 100,000) Among Hispanic or Latino Males in Los Angeles County and the San Francisco Bay Area (2004-2021)
eFigure 3. Delay-Adjusted Incidence Rates of Total Prostate Cancer and Age-Adjusted Prostate Cancer Mortality Rates (per 100,000) Among Non-Hispanic Black Males in Los Angeles County and the San Francisco Bay Area (2004-2021)
eFigure 4. Delay-Adjusted Incidence Rates of Total Prostate Cancer and Age-Adjusted Prostate Cancer Mortality Rates (per 100,000) Among Non-Hispanic White Males in Los Angeles County and the San Francisco Bay Area (2004-2021)

This supplemental material has been provided by the authors to give readers additional information about their work.

© 2025 Van Blarigan EL et al. *JAMA Network Open*.

eTable 1. List of counties by region, California (https://census.ca.gov/regions/)	
**Region**	**Counties**
Superior California	Butte, Colusa, El Dorado, Glenn, Lassen, Modoc, Nevada, Placer, Plumas, Sacramento, Shasta, Sierra, Siskiyou, Sutter, Tehama, Yolo, Yuba
North Coast	Del Norte, Humboldt, Lake, Mendocino, Napa, Sonoma, Trinity
San Francisco Bay Area	Alameda, Contra Costa, Marin, San Francisco, San Mateo, Santa Clara, Solano
Northern San Joaquin Valley	Alpine, Amador, Calaveras, Madera, Mariposa, Merced, Mono, San Joaquin, Stanislaus, Tuolumne
Central Coast	Monterey, San Benito, San Luis Obispo, Santa Barbara, Santa Cruz, Ventura
Southern San Joaquin Valley	Fresno, Inyo, Kern, Kings, Tulare
Inland Empire	Riverside, San Bernardino
Los Angeles County	Los Angeles
Orange County	Orange
San Diego - Imperial	Imperial, San Diego

© 2025 Van Blarigan EL et al. *JAMA Network Open*.

eTable 2. Number of cases and deaths due to prostate cancer in California, by race, ethnicity, and region (2004-2021).[a]			
	**Total prostate cancer cases**	**Cases of distant stage prostate cancer**	**Deaths due to prostate cancer**
	**No. (%)**	**No. (%)**	**No. (%)**
**Overall**	387,636	27,938	58,754
**Age[b]**			
<55	30,713 (7.9)	1,444 (5.2)	815 (1.4)
55 – 69	203,038 (52.4)	10,026 (35.9)	10,045 (17.1)
70 or older	153,884 (39.7)	16,467 (58.9)	47,894 (81.5)
**Race and Ethnicity**			
American Indian, Alaska Native[c]	1,031 (0.3)	90 (0.3)	493 (0.8)
Asian American, Native Hawaiian, Pacific Islander	31,366 (8.1)	2,507 (9.0)	3,979 (6.8)
Hispanic or Latino	66,695 (17.2)	5,482 (19.6)	9,325 (15.9)
Non-Hispanic Black	36,808 (9.5)	3,173 (11.4)	6,401 (10.9)
Non-Hispanic White	238,229 (61.5)	16,571 (59.3)	38,446 (65.4)
Unknown/Other races[3]	13,507 (3.5)	115 (0.4)	110 (0.2)
**Census Region**			
Superior California	36,024 (9.3)	2,834 (10.1)	5,723 (9.7)
North Coast	12,362 (3.2)	1,113 (4.0)	2,100 (3.6)
San Francisco Bay Area	73,834 (19.0)	5,660 (20.3)	9,847 (16.8)
Northern San Joaquin Valley	17,562 (4.5)	1,315 (4.7)	2,884 (4.9)
Central Coast	27,918 (7.2)	1,800 (6.4)	3,710 (6.3)
Southern San Joaquin Valley	19,575 (5.0)	1,431 (5.1)	3,086 (5.3)
Inland Empire	42,584 (11.0)	2,920 (10.5)	6,890 (11.7)
Los Angeles County	91,674 (23.6)	6,112 (21.9)	14,344 (24.4)
Orange County	31,585 (8.1)	1,941 (6.9)	4,433 (7.5)
San Diego - Imperial	34,518 (8.9)	2,812 (10.1)	5,737 (9.8)

[a] Reporting incidence case counts from a registry-specific model that adjusts numbers for delays in reporting.
[b] One case was missing age at diagnosis.

© 2025 Van Blarigan EL et al. *JAMA Network Open*.

[c] American Indian, Alaska Native race is only available for delay-adjusted incident cases with residence at diagnosis in a Purchased/Referred Care Delivery Area (PRCDA)-county. American Indian, Alaska Native people diagnosed with prostate cancer in a non-PRCDA county are classified as unknown/other race (the number of people in this group is not identifiable). This limitation does not apply to deaths.

eTable 3. Trends in prostate cancer delay-adjusted incidence rates in California, overall and by stage, age, race and ethnicity, and region, 2004-2021.

	Trend 1		Trend 2		Trend 3		Trend 4	
	**Years**	**APC (95% CI)**	**Years**	**APC (95% CI)**	**Years**	**APC (95% CI)**	**Years**	**APC (95% CI)**
**Overall**	2004-2010	-2.0 (-3.3, 0.1)	2010-2014	-9.7 (-13.2, -7.0)	2014-2021	2.6 (1.1, 4.5)	NA	NA
**Stage**								
Localized	2004-2011	-3.7 (-4.7, -1.8)	2011-2014	-13.6 (-15.8, -9.2)	2014-2021	1.0 (-0.4, 3.2)	NA	NA
Regional	2004-2010	-0.8 (-2.5, 4.2)	2010-2013	-9.8 (-12.8, -4.3)	2013-2021	3.0 (1.2, 6.2)	NA	NA
Distant	2004-2011	-1.3 (-2.7, -0.3)	2011-2021	6.7 (6.2, 7.3)	NA	NA	NA	NA
**Age**								
<55	2004-2011	0.6 (-0.6, 2.4)	2011-2014	-14 (-16.5, -9.1)	2014-2021	-1.8 (-3.6, 1.5)	NA	NA
55 – 69	2004-2011	-1.9 (-2.8, -0.2)	2011-2014	-11.6 (-13.6, -7.5)	2014-2021	2.3 (0.9, 4.2)	NA	NA
70 or older	2004-2010	-3.4 (-4.5, -0.9)	2010-2014	-9.5 (-12.5, -6.8)	2014-2021	3.8 (2.5, 5.3)	NA	NA
**Race and Ethnicity**								
AIAN[a]	2004-2018	-2.9 (-7.3, -1.4)	2018-2021	11.5 (-0.8, 18.2)	NA	NA	NA	NA
AANHPI	2004-2011	-3.0 (-4.1, -1.3)	2011-2014	-13.4 (-15.4, -8.9)	2014-2021	3.7 (2.4, 5.3)	NA	NA
Hispanic or Latino	2004-2010	-2.8 (-3.8, -0.5)	2010-2014	-8.8 (-11.9, -6.5)	2014-2021	0.6 (-0.7, 2.4)	NA	NA
Non-Hispanic Black	2004-2010	-1.4 (-2.8, 0.9)	2010-2015	-8.9 (-13.1, -6.7)	2015-2021	4.6 (2.5, 7.2)	NA	NA
Non-Hispanic White	2004-2010	-2.1 (-3.5, 0.6)	2010-2014	-9.8 (-13.5, -6.8)	2014-2021	3.1 (1.4, 5.3)	NA	NA
**Region**								
Superior California	2004-2007	4.3 (-0.6, 12.6)	2007-2015	-6.8 (-9.9, -5.6)	2015-2021	2.4 (-0.1, 6.2)	NA	NA
North Coast	2004-2007	9.7 (2.8, 20.8)	2007-2015	-9.3 (-12.9, -7.6)	2015-2021	5.9 (2.1, 11.4)	NA	NA
San Francisco Bay Area	2004-2011	-1.9 (-3.5, 1.2)	2011-2014	-14.9 (-18.1, -8.3)	2014-2021	2.8 (0.5, 6.8)	NA	NA
Northern San Joaquin Valley	2004-2007	3.8 (0.4, 9.9)	2007-2011	-4.6 (-10.7, -1.7)	2011-2014	-14.4 (-16.4, 1.8)	2014-2021	4.8 (3.3, 6.6)
Central Coast	2004-2010	-2.3 (-4.6, 4.3)	2010-2014	-11 (-17.3, -6.1)	2014-2021	7.2 (4.4, 11.8)	NA	NA
Southern San Joaquin Valley	2004-2016	-4.3 (-8.8, -3.0)	2016-2021	3.5 (-1.7, 12.2)	NA	NA	NA	NA
Inland Empire	2004-2010	-2.0 (-3.4, 2.9)	2010-2014	-6.5 (-9.6, -3.7)	2014-2021	0.4 (-1.1, 3.3)	NA	NA
Los Angeles County	2004-2010	-3.5 (-5.4, 3.4)	2010-2015	-7.4 (-12.7, -3.4)	2015-2021	2.7 (-0.2, 7.8)	NA	NA

© 2025 Van Blarigan EL et al. *JAMA Network Open*.

Orange County	2004-2011	-3.3 (-6.6, 7.7)	2011-2014	-8.8 (-13, 3.9)	2014-2021	2.7 (-3.7, 8.9)	NA	NA
San Diego - Imperial	2004-2008	0.5 (-2.3, 7.2)	2008-2014	-6.9 (-12.1, -5)	2014-2021	2.9 (1.0, 5.7)	NA	NA

Abbreviations: APC, annual percent change; AANHPI, Asian American, Native Hawaiian, Pacific Islander; AIAN, American Indian, Alaska Native; CI, confidence interval.
[a] Analyses among AIAN individuals restricted to Purchased/Referred Care [PRC] Delivery Area (PRCDA)-counties.

eTable 4. Trends in delay-adjusted incidence rates for distant stage prostate cancer in California, overall and by age, race and ethnicity, and region (2004-2021).

	Trend 1		Trend 2		Trend 3	
	**Years**	**APC (95% CI)**	**Years**	**APC (95% CI)**	**Years**	**APC (95% CI)**
**Overall**	2004-2011	-1.3 (-2.7, -0.3)	2011-2021	6.7 (6.2, 7.3)	NA	NA
**Age, years**						
<55	2004-2021	1.8 (0.7, 3.1)	NA	NA	NA	NA
55 – 69	2004-2012	0.3 (-1.6, 1.6)	2012-2021	6.9 (6.0, 8.1)	NA	NA
70 or older	2004-2011	-1.9 (-3.9, -0.4)	2011-2021	7.1 (6.3, 8.1)	NA	NA
**Race and Ethnicity**[a]						
AANHPI	2004-2011	-3.0 (-17.6, 1.9)	2011-2021	6.5 (4.2, 13.4)	NA	NA
Hispanic or Latino	2004-2007	-6.8 (-12.4, -2.6)	2007-2014	0.6 (-0.9, 3.7)	2014-2021	8.0 (6.9, 9.5)
Non-Hispanic Black	2004-2013	-0.5 (-4.8, 1.7)	2013-2021	7.4 (5.1, 11.4)	NA	NA
Non-Hispanic White	2004-2010	-1.6 (-3.4, -0.3)	2010-2021	6.9 (6.4, 7.5)	NA	NA
**Region**						
Superior California	2004-2013	2.7 (-8.3, 10.9)	2013-2021	7.0 (4.2, 12.9)	NA	NA
North Coast	2004-2010	-6.0 (-30.9, 4.4)	2010-2021	8.0 (3.0, 22.8)	NA	NA
San Francisco Bay Area	2004-2014	1.1 (-8.1, 3.4)	2014-2021	8.0 (4.6, 15.4)	NA	NA
Northern San Joaquin Valley	2004-2010	-1.4 (-12.7, 3.2)	2010-2021	6.8 (5.2, 10.6)	NA	NA
Central Coast	2004-2013	2.1 (-15.3, 22.5)	2013-2021	9.1 (4.3, 19.1)	NA	NA
Southern San Joaquin Valley	2004-2021	2.3 (0.4, 4.6)	NA	NA	NA	NA
Inland Empire	2004-2011	-4.8 (-8.9, -2.1)	2011-2021	7.3 (5.8, 9.4)	NA	NA
Los Angeles County	2004-2011	-2.4 (-5.3, -0.4)	2011-2021	6.3 (5.2, 7.8)	NA	NA
Orange County	2004-2013	0.2 (-8.2, 2.9)	2013-2021	7.8 (5.0, 13.7)	NA	NA
San Diego - Imperial	2004-2010	-2.5 (-16.2, 2.8)	2010-2021	7.3 (5.4, 12.0)	NA	NA

Abbreviations: APC, annual percent change; AANHPI, Asian American, Native Hawaiian, Pacific Islander; CI, confidence interval.
[a] Annual percent change values could not be calculated for American Indian, Alaska Native people due to small numbers (≤16 cases annually or population less than 10,000).

© 2025 Van Blarigan EL et al. *JAMA Network Open*.

**eTable 5. Trends in prostate cancer mortality rates in California, overall and by age, race and ethnicity, and region (2004-2021).**						
	**Trend 1**		**Trend 2**		**Trend 3**	
	**Years**	**APC (95% CI)**	**Years**	**APC (95% CI)**	**Years**	**APC (95% CI)**
**Overall**	2004-2012	-2.6 (-4.5, -1.7)	2012-2021	0.1 (-0.6, 1.6)	NA	NA
**Age, years**						
<55	2004-2021	0.0 (-1.4, 1.6)	NA	NA	NA	NA
55-69	2004-2021	-0.9 (-1.4, -0.4)	NA	NA	NA	NA
70+	2004-2012	-2.8 (-4.2, -2.0)	2012-2021	0.2 (-0.5, 1.3)	NA	NA
**Race and ethnicity**[a]						
AANHPI	2004-2009	-5.4 (-15.7, -1.4)	2009-2021	-0.1 (-1.1, 5.1)	NA	NA
Hispanic or Latino	2004-2014	-2.5 (-6.8, -1.4)	2014-2021	0.4 (-1.1, 5.7)	NA	NA
NH Black	2004-2021	-1.5 (-2.3, -0.7)	NA	NA	NA	NA
NH White	2004-2012	-2.3 (-4.3, -1.4)	2012-2021	0.5 (-0.2, 2.1)	NA	NA
**Region**						
Superior California	2004-2016	-1.4 (-4.3, -0.6)	2016-2021	1.8 (-0.4, 7.6)	NA	NA
North Coast	2004-2011	-4.4 (-14.5, -1.0)	2011-2021	0.6 (-1.9, 10.6)	NA	NA
San Francisco Bay Area	2004-2014	-2.4 (-4.7, -1.6)	2014-2021	0.3 (-0.9, 3.9)	NA	NA
Northern San Joaquin Valley	2004-2011	-4.5 (-15.5, -0.9)	2011-2016	5.4 (0.8, 15.9)	2016-2021	-4.9 (-17.2, -1.2)
Central Coast	2004-2021	-1.4 (-2.3, -0.4)	NA	NA	NA	NA
Southern San Joaquin Valley	2004-2014	-2.9 (-10.5, -0.9)	2014-2021	1.2 (-1.7, 11.5)	NA	NA
Inland Empire	2004-2012	-3.1 (-12.0, 3.2)	2012-2021	0.7 (-7.6, 9.8)	NA	NA
Los Angeles County	2004-2021	-1.2 (-1.8, -0.5)	NA	NA	NA	NA
Orange County	2004-2006	-9.5 (-13.2, -2.1)	2006-2021	-1.1 (-1.5, 0.2)	NA	NA
San Diego - Imperial	2004-2021	-0.7 (-1.3, 0.0)	NA	NA	NA	NA

Abbreviations: APC, annual percent change; AANHPI, Asian American, Native Hawaiian, Pacific Islander; CI, confidence interval.
[a] Annual percent change values could not be calculated for American Indian, Alaska Native people due to small numbers (≤16 cases annually or population less than 10,000).

© 2025 Van Blarigan EL et al. *JAMA Network Open*.

eFigure 1. Incidence rates of distant prostate cancer and prostate cancer mortality rates (per 100,000) for San Francisco Bay Area and Los Angeles County among Asian American, Native Hawaiian, Pacific Islander males (2004-2021)

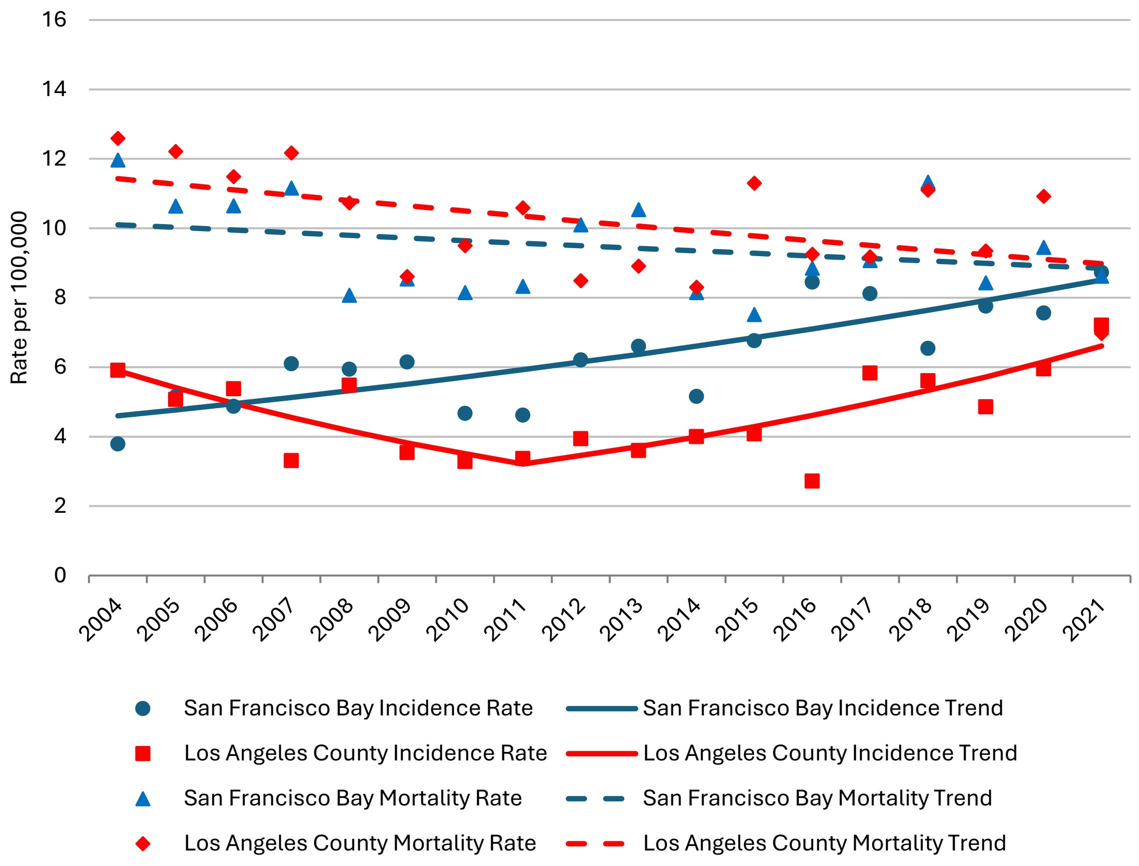

© 2025 Van Blarigan EL et al. *JAMA Network Open*.

eFigure 2. Incidence rates of distant prostate cancer and prostate cancer mortality rates (per 100,000) for San Francisco Bay Area and Los Angeles County among Hispanic males (2004-2021)

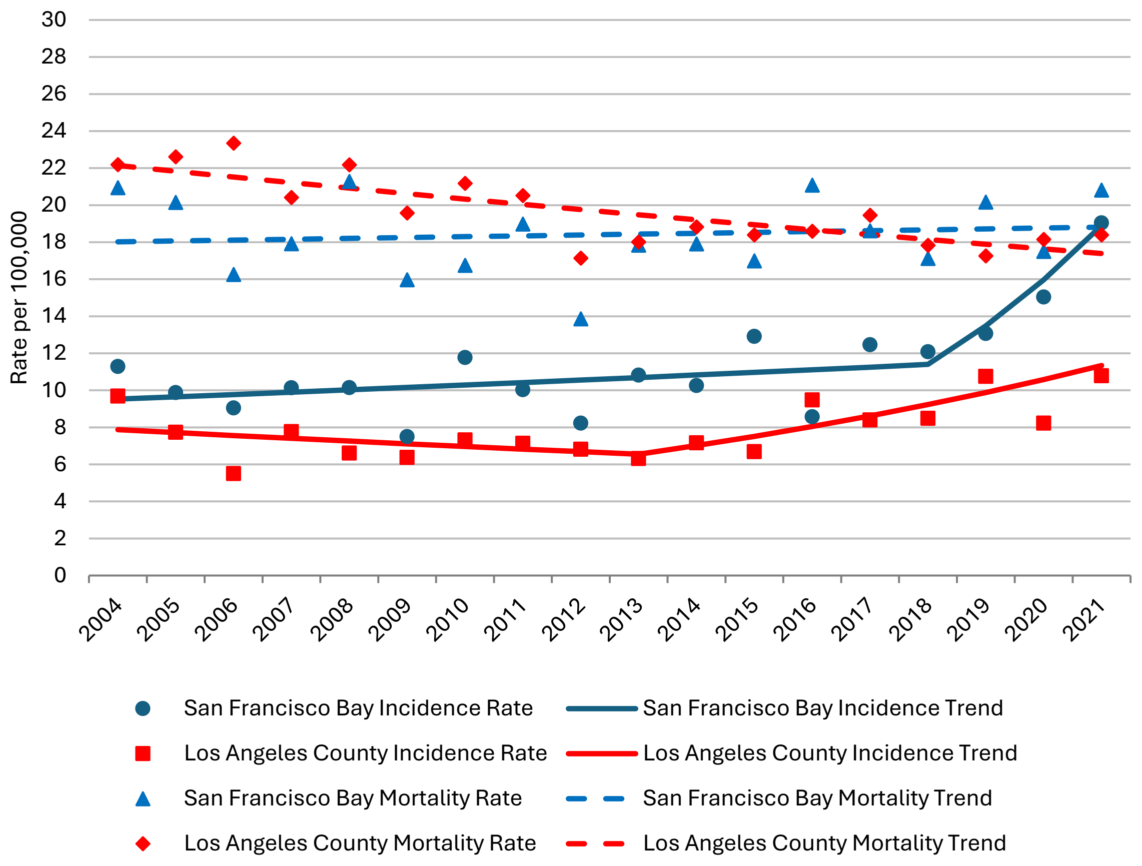

© 2025 Van Blarigan EL et al. *JAMA Network Open*.

eFigure 3. Incidence rates of distant prostate cancer and prostate cancer mortality rates (per 100,000) for San Francisco Bay Area and Los Angeles County among Non-Hispanic Black males (2004-2021)

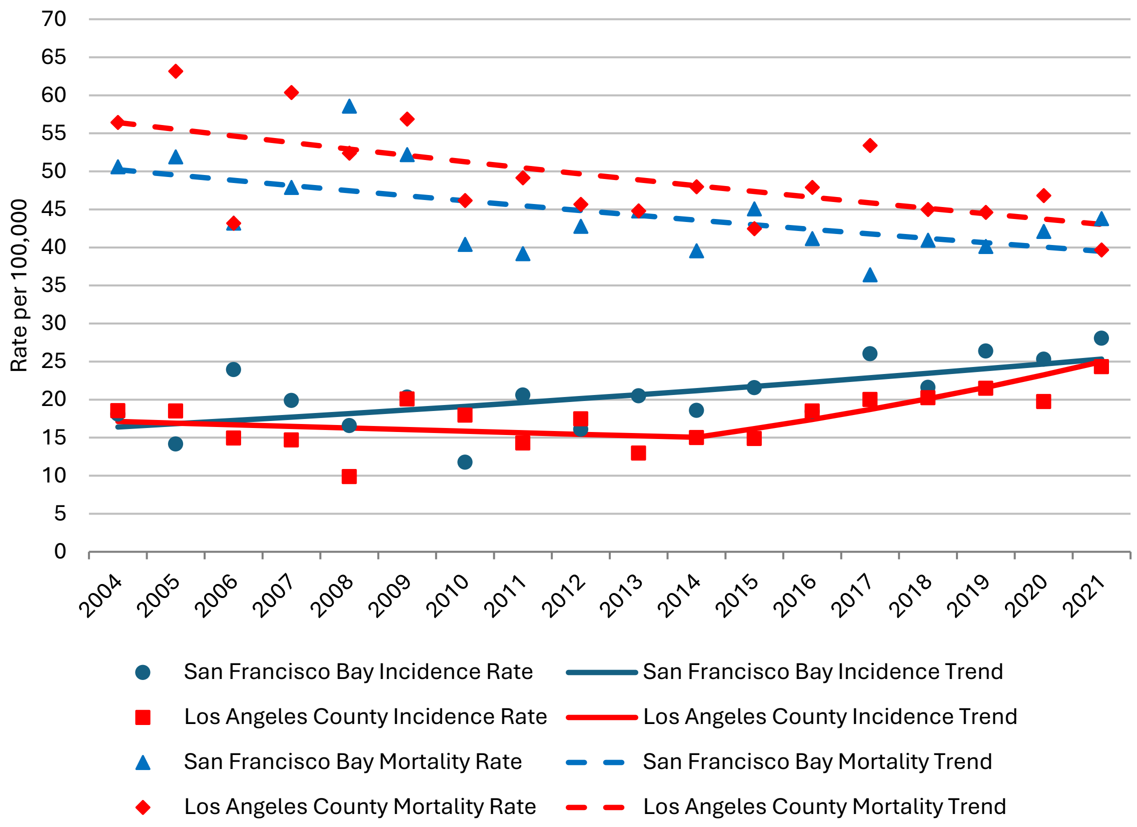

© 2025 Van Blarigan EL et al. *JAMA Network Open*.

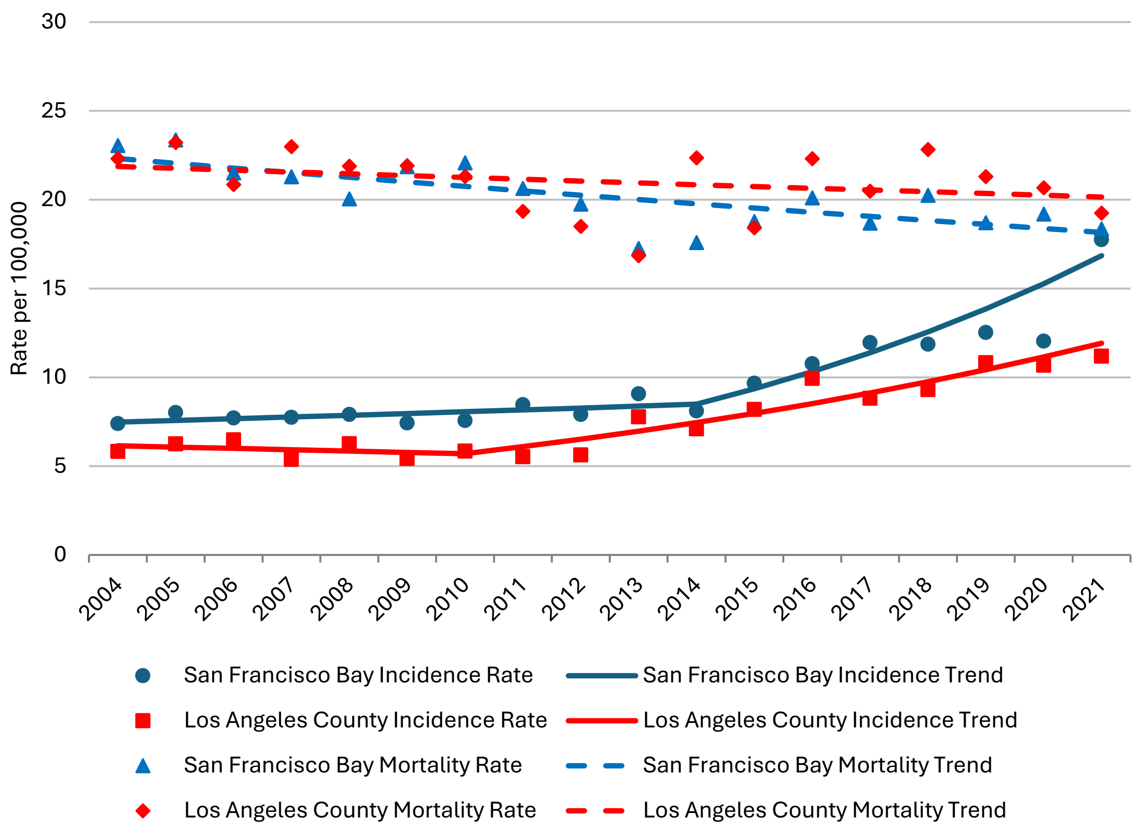

eFigure 4. Incidence rates of distant prostate cancer and prostate cancer mortality rates (per 100,000) for San Francisco Bay Area and Los Angeles County among Non-Hispanic White males (2004-2021)

© 2025 Van Blarigan EL et al. *JAMA Network Open*.
