# Supplementary material for: Trends in Prostate Cancer Incidence and Mortality Rates
Source: JAMA Netw Open. 2025 Jan 27;8(1):e2456825. doi: 10.1001/jamanetworkopen.2024.56825 (PMC11774093; doi:10.1001/jamanetworkopen.2024.56825)
Supplement: Supplement 2. — Data Sharing Statement [file jamanetwopen-e2456825-s002.pdf]

Data Sharing Statement

Van Blarigan. Trends in Prostate Cancer Incidence and Mortality Rates. *JAMA Netw Open*. Published January 27, 2025. doi:10.1001/jamanetworkopen.2024.56825

Data
Data available: Yes
Data types: The data generated in this study are publicly available from the Surveillance, Epidemiology, and End Results (SEER) Program (www.seer.cancer.gov) and the California Cancer Registry (https://www.ccrcal.org/retrieve-data/data-for-the-public/). The mortality data used in this study are provided in a SEER*Stat database produced by the CCR, available with permission from the CCR.
When available: beginning date: 04-16-2024

Supporting Documents
Document types: Statistical/analytic code
How to access documents: meg.mckinley@ucsf.edu
When available: With publication

Additional Information
Who can access the data: Anyone requesting the data
Types of analyses: For any purpose
Mechanisms of data availability: Without investigator support
